# Biosensors Based on Stanniocalcin-1 Protein Antibodies Thin Films for Prostate Cancer Diagnosis

**DOI:** 10.3390/bios13110981

**Published:** 2023-11-10

**Authors:** Renato Ferreira, Paulo A. Ribeiro, Adelino V. M. Canário, Maria Raposo

**Affiliations:** 1 Laboratory of Instrumentation, Biomedical Engineering and Radiation Physics (LIBPhys-UNL), Department of Physics, NOVA School of Science and Technology, Universidade NOVA de Lisboa, 2829-516 Caparica, Portugal; rsa.ferreira@campus.fct.unl.pt (R.F.); pfr@fct.unl.pt (P.A.R.); 2 Centre of Marine Sciences (CCMAR), University of Algarve, Campus de Gambelas, 8005-139 Faro, Portugal; acanario@ualg.pt

**Keywords:** prostate cancer, electronic tongue, stanniocalcin, STC1, layer-by-layer films, polyelectrolytes, impedance spectroscopy, principal component analysis

## Abstract

Prostate cancer is one of the most prevalent tumors in men, accounting for about 7.3% of cancer deaths. Although there are several strategies for diagnosing prostate cancer, these are only accurate when the tumor is already at a very advanced stage, so early diagnosis is essential. Stanniocalcin 1 (STC1) is a secreted glycoprotein, which has been suggested as a tumor marker as its increased expression is associated with the development and/or progression of different types of malignant tumors. In this work, an electronic tongue (ET) prototype, based on a set of four sensors prepared from thin films that included STC1 antibodies for detecting prostate cancer, was developed. In the preparation of the thin films, polyelectrolytes of polyallylamine hydrochloride, polystyrene sulfonate of sodium and polyethyleneimine, and the biomolecules chitosan, protein A, and STC1 antibody were used. These films were deposited on quartz lamellae and on solid supports using layer-on-layer and self-assembly techniques. The deposition of the films was analyzed by ultraviolet-visible spectroscopy, and the detection of STC1 in aqueous solutions of PBS was analyzed by impedance spectroscopy. The impedance data were statistically analyzed using principal component analysis. The ETs formed by the four sensors and the three best sensors could detect the antigen at concentrations in the range from 5 × 10^−11^ to 5 × 10^−4^ M. They showed a linear dependence with the logarithm of the antigen concentration and a sensitivity of 5371 ± 820 and 4863 ± 634 per decade of concentration, respectively. Finally, the results allow us to conclude that this prototype can advance to the calibration phase with patient samples.

## 1. Introduction

Prostate cancer is one of the tumors that most affect men, responsible for about 7.3% of cancer deaths, and one of the top five tumors with the highest mortality rate [1,2]. However, it has a good prognosis and is usually treatable. Patient survival can increase to almost 100% of patients over 5 years when detected early. However, the prognosis is poor with late detection, decreasing to 31% survival during the same period because, even without significant symptoms, it can develop metastases in bone or other organs, drastically increasing the mortality rate [1,3,4]. Depending on the extent/severity of the cancer, patient characteristics, age, and personal preference, several treatment strategies for prostate cancer, such as chemotherapy, radiotherapy, surgery, hormone therapy, active surveillance, or a combination, are commonly used [5]. However, if the cancer is already at an advanced stage, some of these strategies may fail, thus making early diagnosis extremely important for survival. The techniques commonly used to diagnose prostate cancer are rectal examination, tissue biopsy, and analysis of prostate-specific antigen (PSA) [6]. Although widely used, the rectal examination, which consists of the analysis of the changes in the prostate’s texture, size, or shape that may indicate the presence of cancer, has a low sensitivity (18–22%) and specificity, making a small contribution to reducing the mortality rate [7]. PSA is a prostate cancer biomarker, as it is only secreted by the prostate, and its expression tends to increase in the presence of cancer [6]. A blood PSA concentration above 4 ng/mL corresponds to a positive diagnosis. However, other conditions such as ageing, benign prostatic hyperplasia (BPH), and prostatitis can increase PSA production and false positives [8]. In addition, several patients with a positive diagnosis of prostate cancer have PSA values below the reference value. PSA does not distinguish whether the tumor is in a proliferative, slow-growing, or invasive phase, thus resulting in extensive and aggressive treatments that are unnecessary and affect the patients’ quality of life on a psychological and physical level [9]. To overcome the limitations above, new prostate cancer biomarkers have been studied. When tumors grow, they release various cells, proteins, and metabolites that are directly related to the stages of the disease and that can be used as diagnostic biomarkers [10,11]. Several non-PSA biomarkers, such as RNA, glycoproteins, proteins, and the *EN2* gene, are available. However, only a few shows good results [1]; therefore, developing new prostate cancer sensors is necessary. Recent studies revealed that the secretion of glycoprotein stanniocalcin-1 (STC1) is associated with the development and progression of different types of malignant tumors, an observation that has led to the proposal that it could be used as a tumor marker for prostate cancer [12,13,14]. These studies suggest that sensors prepared with STC1 antibodies may be an exciting new approach for STC1 detection and, consequently, prostate cancer detection.

One of the approaches for developing sensors is the concept of an electronic tongue, consisting of an array of sensors whose electrical signals, after mathematical treatment, enable the distinction of various aqueous matrices [15]. The electronic tongue has been successfully used to detect the concentration of molecules in complex matrices, namely molecules such as triclosan [16] and estrogen [17] at concentrations near 10^−15^ M, making this method valuable for detecting low concentrations of the STC1 antigen. In this work, the electronic tongue concept with an array of sensors prepared with thin films of STC1 antibodies from a fish was used to develop a sensorial device to detect STC1 antigen and, possibly, prostate cancer in the future. The sensors that allow for STC1 antigen detection were based on thin films prepared onto a solid support with gold interdigitated electrodes (IDEs), using layer-by-layer (LbL) and self-assembly thin film preparation techniques. The antigen detection was performed by impedance spectroscopy with sensors immersed in solutions with different antigen concentrations. The electrical properties of these films were analyzed together and individually by principal component analysis (PCA) to define the ability of the films obtained to be effective sensors of STC1 concentration.

## 2. Materials and Methods

### 2.1. Developed Sensors

**Figure 1:**
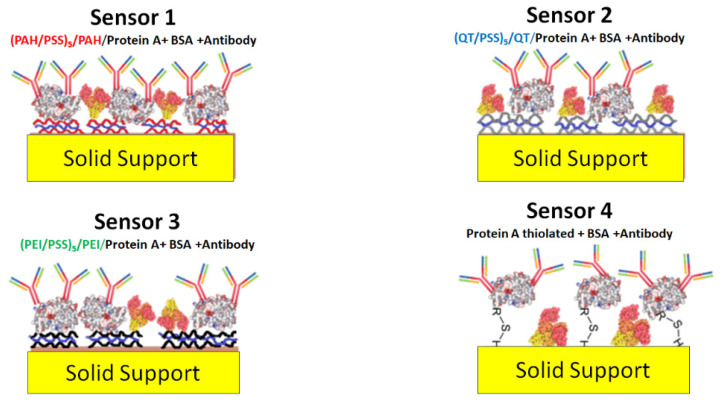
Schema of the different proposed sensors.

To develop sensors in which STC1 antibody layers were deposited on solid supports with gold IDEs, a four-sensors approach, S1 to S4, has been developed. In three of these different sensors, S1 to S3, layer-by-layer (LbL) structures were prepared with polyelectrolytes poly allylamine hydrochloride (PAH), polysodium polystyrene sulfonate (PSS), and polyethyleneimine (PEI) and chitosan to be used as cushions of the STC1 antibody. The S1, S2, and S3 cushion’s structures are (PAH/PSS)_5_/PAH, (CT/PSS)_5_/CT, and (PEI/PSS)_5_/PEI, respectively, while for S4, the STC1 antibody layer was adsorbed on the gold electrodes without any deposited cushion. The antibody layer also includes protein A and bovine serum albumin (BSA), as explained in Section 2.3. Briefly, sensors S1 to S3 are composed of (PAH/PSS)_5_/PAH/(protein A + BSA + antibody), (CT/PSS)_5_/CT/(protein A + BSA + antibody), and (PEI/PSS)_5_/PEI/(protein A + BSA + antibody), respectively, while S4 is composed by protein A thiolated + BSA + antibody. Figure 1 presents the schemes for these sensors.

### 2.2. Cushion’s Preparation

#### 2.2.1. Cushion’s Materials

**Figure 2:**
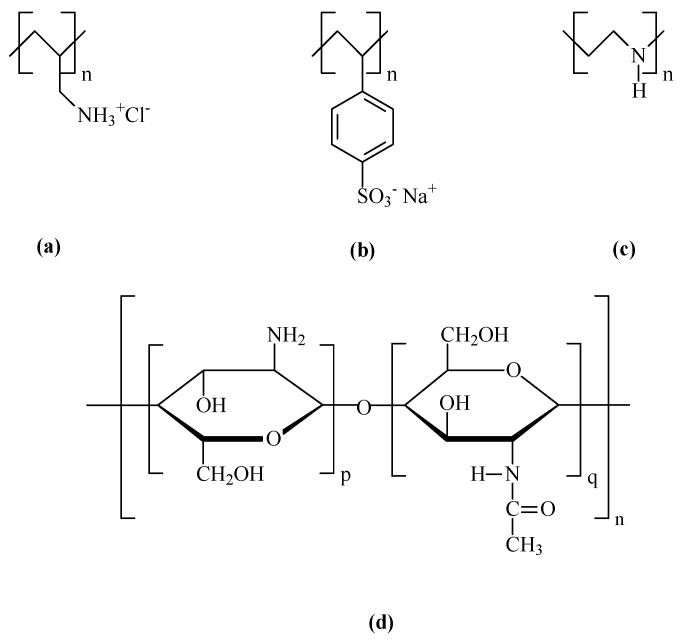
Molecular structures of the used polyelectrolytes, (**a**) PAH (Cas number: 71550-12-4); (**b**) PSS (Cas number: 25704-18-1); (**c**) PEI (Cas number: PEI—49553-93-7); and (**d**) Chitosan (Cas number: 9012-76-4).

The chemicals used in this work were purchased from Sigma-Aldrich (St. Louis, MO, USA). Polyelectrolytes as poly allylamine hydrochloride (PAH), poly sodium polystyrene sulfonate (PSS), and polyethyleneimine (PEI), with average molecular weights of 17,500, 70,000, and 75,000 g/mol, respectively, and chitosan (CT) a non-toxic, odorless, biocompatible, and biodegradable biopolymer with a molecular weight between 1.5 × 10^6^ and 1.8 × 10^6^ g/mol obtained from the natural polymer chitin, were used in this work. The molecular structures of these polyelectrolytes and chitosan are represented in Figure 2.

#### 2.2.2. Polyelectrolyte Solutions

PAH, PSS, and PEI aqueous solutions with a monomeric concentration of 10^−3^ M were prepared by dissolving 0.0234, 0.0395, and 0.0193 g, respectively, each in 25 mL of ultrapure water with a resistivity of 19.8 MΩcm and pH of 5.6 supplied by a waterMax system Diwer Technologies (Austin, TX, USA). Chitosan solutions (1% *w*/*v*) in 0.05 M acetic acid were prepared. [18]. When dissolved in acetic acid, chitosan becomes a polycation by the chemical reaction of protonation of chitosan in acetic acid and polycation formation.

#### 2.2.3. Layer-by-Layer Thin Films

The sensing devices are based on thin films prepared by layer-by-layer (LbL) and self-assembly techniques [19]. These techniques, associated with electrostatic and chemical interactions, consist of the adsorption of molecular layers at the solid/liquid interface onto solid supports, such as quartz and ceramic supports, with the interdigitated electrodes.

Quartz solid supports were used to study the formation of the LbL films and the effect of phosphate-buffered saline (PBS) solutions on these films. These supports were previously hydrophilized in piranha solutions prepared with 75 mL of sulfuric acid and 25 mL of hydrogen peroxide for 1 h and washed 5 times in ultrapure water to remove residues of the piranha solution. This hydrophilization step removes all organic residues from the substrates and makes them rougher and richer in the OH groups for better adsorption of the polycationic molecules. Quartz solid supports were stored in ultrapure water until the thin films were produced, keeping them negatively charged and thus allowing for a better adsorption of positively charged polyelectrolytes.

The sensor devices consist of a solid ceramic support with interdigitated gold electrodes deposited on one of the surfaces and purchased from Metrohm dropsense (DS) (Oviedo, Asturias, Spain) [20]. According to DS, the interdigitated electrodes have a distance between electrodes W and width of electrodes S of 200 µm. From these values, the maximum reach distance of the electric field (D) created by the positive and negative poles was estimated as D = (S + W) × 2. This value must be considered when depositing the layers of thin films on the interdigitated electrodes to avoid sensor sensitivity loss.

For the production of the (PAH/PSS)_n_, (QT/PSS)_n_, and (PEI/PSS)_n_ LbL thin films, being n the number of bilayers, the quartz supports were immersed in the polycationic aqueous solution for 1 min, then in ultrapure water for 5 sec to remove non-adsorbed molecules residues, and dried with compressed nitrogen. After forming this first monolayer, the solid supports were immersed in the anionic solution for 1 min, followed by ultrapure water for 5 sec, and dried with nitrogen. This formed the first bilayer. This process was then repeated n times until the formation of n bilayers. These films were used to adsorb protein A + BSA and antibodies by the self-assembly technique [21,22], allowing for the spontaneous formation of molecular assemblies by chemosorption through the immersion of an appropriate substrate in a solution of active surfactant in an organic solvent.

### 2.3. Antibody Layer

#### 2.3.1. Immobilization of Protein A

One of the most critical steps in constructing a sensor is the biofunctionalization of the surface for the correct identification of the analyte under study. The proper immobilization of antibodies on the sensors’ surface directly influences the sensor’s final performance. Protein A is found in the cell wall of *Staphylococcus aureus* bacteria and, together with G protein, has been used in the correct immobilization of antibodies on the surface of sensors, increasing the sensor sensitivity and specificity toward antigens and the performance of the sensor [23,24].

The advantage of using protein A over protein G is that it has more antibody-binding domains. Based on the study carried out by Carosseli et al. [25], the use of protein A is essential, as it can bind the crystallizable fractions of antibodies, the regions that bind to the surface of the sensors, making the fractions binding to the antigen (Fab) available, thus providing greater and better targeting of the antibodies and increasing the sensitivity and specificity of the sensor. The disadvantage of using proteins to increase the orientation/sensitivity of sensors is that there is a smaller number of antibodies adsorbed per unit area and possible changes in their conformation.

The protein A solution was prepared in phosphate-buffered saline (PBS) according to Caroselli et al. [25]. A buffer solution is essential because it provides a stable pH of around 8, close to the ideal binding pH between protein A and antibodies, thus maximizing antibody binding to the substrate. A stock solution of PBS 1 M was prepared, from which 0.1 M PBS dilutions were prepared. Protein A-Sepharose (0.5 mg) from *Staphylococcus aureus* was dissolved in 1 mL of 0.1 M PBS, obtaining a stock solution of protein A with a concentration of 0.5 mg/mL. The protein A mother solution was again diluted to 10 µg/mL protein A.

A 0.02% (*w*/*v*) bovine serum albumin (BSA) solution in 0.05 M PBS was used to block sites not adsorbed to protein A. To increase the binding affinity of protein A to gold, amine groups were converted to thiol groups using 2-iminothiolane. This change was made by adding 0.0014 g of 2-iminothiolane to a new 10 µg/mL protein A solution [26].

#### 2.3.2. Immobilization of Antibody

The rabbit polyclonal antibody was produced against a peptide from the N-terminal region of STC1A from pufferfish (*Tetraodon nigroviridis*), an isoform of human STC1 [27]. For the preparation of the antibody solution, 100 µL of serum was diluted in 200 mL of 0.01 M PBS with 0.02% (*w*/*v*) sodium azide and stored at 4 °C. Antibody solutions were prepared in the concentration range from 5 × 10^−4^ to 5 × 10^−11^ M.

#### 2.3.3. Antigen Solutions

The antigen STC1A was isolated from the sea bass (*Dicentrarchus labrax*) corpuscles of Stannius [28]. The STC1A solution was diluted in a Tris-HCl buffer solution (25 mM, pH—8) to a 30 µg/mL concentration and stored at −80 °C until use.

### 2.4. Methods of Characterization

#### 2.4.1. Optical and Electrical Characterization

The absorbance of the LbL films prepared onto quartz solid supports was analyzed between 190 and 900 nm on a double-beam Shimadzu UV—2101 PC UV–vis spectrophotometer (Shimadzu Europa GmbH, Duisburg, Germany).

A Solartron 1260 impedance analyzer coupled to a 1296A dielectric interface (Solartron Analytical, Farnborough, Hampshire, UK), with a frequency between 1 Hz and 1 × 10^6^ Hz, was used to measure the impedance, capacitance, and resistance of sensors [29]. The applied voltage (AC) was 25 mV to avoid damage to the IDEs when immersed in aqueous solutions because, according to Zagalo et al. [30], electrical voltages above this value can destroy the electrodes. Below this value, the IDEs start to lose sensitivity. All measurements began with a 30 s delay to allow the system to stabilize and were repeated in a 3-times cycle. To control parameters such as voltage (AC and DC), frequency range, and number of measurements, among others, the SMaRT Impedance software (Solartron Analytical, Farnborough, Hampshire, UK) was used.

#### 2.4.2. Data Treatment

Spectroscopic impedance generates a large multivariate dataset. Principal components analysis (PCA) is a statistical method used to reduce the dimensionality of the extensive dataset while preserving the most relevant information in the data, transforming a set of correlated data into a smaller set of uncorrelated data that maximizes the variance of the system, that is, preserving as much of the variability as possible—the main components of the system. The objective is to find new variables that are linear functions of the initial variables, maximize the variability between them, and ensure that are correlated. The PCA also aims to eliminate some variables that characterize the system but have little weight or statistical information [31,32]. One starts by finding the direction with the most statistical information and the greatest variability, defined as the first principal component. The second principal component is the one with the smallest variance orthogonal to the first. Each additional component is found following the same procedure. Two-dimensional graphics are obtained to visualize the different categories grouped according to their similarities or differences.

A PCA was performed using OriginPro 2021b. To make the results more robust, impedance and loss tangent data from all sensors and antigen concentrations were used in a frequency range from 1 to 30 Hz.

## 3. Results

### 3.1. Development of Antibody’s Cushions

#### 3.1.1. Cushion’s Characterization

**Figure 3:**
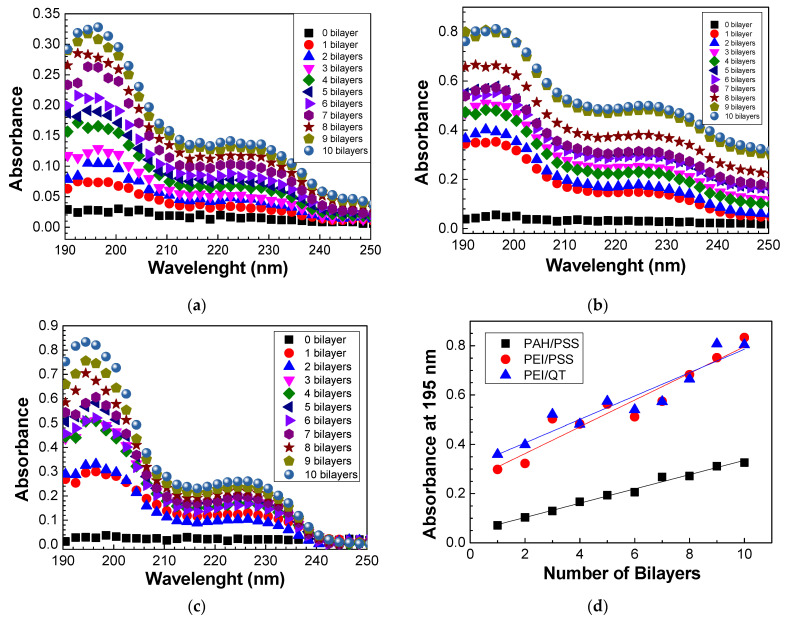
Absorbance spectra of the (**a**) (PAH/PSS)_20_, (**b**) (PEI/PSS)_20_, and (**c**) (QT/PSS)_20_ LbL films. (**d**) The evolution of absorbance at 195 nm of the different LbL films with the number of bilayers.

Firstly, preparing and characterizing the PAH/PSS, QT/PSS, and PEI/PSS LbL films was necessary to develop the sensors. As PSS and QT molecules absorb in the visible wavelength region, the growth of these films on the quartz lamellae was analyzed by UV–vis spectroscopy. The UV–vis spectra of the PAH/PSS, QT/PSS, and PEI/PSS LbL films for the different numbers of adsorbed bilayers are represented in Figure 3a–c, respectively.

From the spectra obtained in Figure 3a–c, it is possible to analyze the growth of PAH/PSS, QT/PSS, and PEI/PSS thin films as well as the increase in absorbance as the number of bilayers increases. In the case of PAH/PSS films, as seen in Figure 3a, there are two absorption bands whose peaks are well-defined at 195 and 227 nm. The absorption band whose peak has a maximum intensity at 227 nm is due to the presence of the Na^+^ ion in the PSS, as indicated in Figure 3b. PAH has no absorption in the visible region, having an absorption band around 150 nm due to the electronic transitions of the NH_3_^+^ group [33], and is not detected in this wavelength region. However, because the growth of thin films through the LbL technique is mainly caused by electrostatic interactions between the opposite electrical charges of the ionic groups in the two polyelectrolytes, it is these interactions that lead to the adsorption of a layer on the oppositely charged polyelectrolyte with the formation of the various bilayers. Therefore, for PSS to be present in the quartz lamella, PAH must also be adsorbed on it, as PSS has anionic groups while PAH has cationic groups. The absorption band with a maximum peak at 195 nm is associated with the π-π* transitions of the PSS benzene group, whose peaks are at 186 nm and 215 nm [33].

The spectra of the QT/PSS films (Figure 3b) demonstrate similar behavior as PAH/PSS thin films. However, for the same number of bilayers, the absorbance of QT/PSS films is higher than that of PAH/PSS films. There can be two main justifications for this fact. The first is the degree of ionization. Polyelectrolytes are classified as strong or weak polyelectrolytes according to their degree of ionization. Weak polyelectrolytes are pH-sensitive, while strong polyelectrolytes are less sensitive. The pH controls the degree of ionization, which is directly related to the dissociation constant and the conformation of the polyelectrolytes’ molecules. Table 1 lists the degree of ionization of polyelectrolytes in the pH range used in this work. The lower the degree of ionization, the greater the adsorption, regardless of the nature of the polyelectrolyte, but other factors such as roughness, intensity of electrostatic forces, and competition for adsorption sites must be considered [34]. The second justification is related to chitosan, which, when dissolved in acetic acid, forms NH_3_ groups absorbing at a wavelength between 200 nm and 230 nm [35] so that the absorbance spectrum of (QT/PSS) this region represents the sum of the chitosan and PSS contributions.

Finally, the spectra of the films of (PEI/PSS) with different bilayers (Figure 3c) again show the presence of peaks at 195 nm and 227 nm due to the presence of PSS on the quartz lamella, given that PEI has no visible absorption band. It is noted that (PEI/PSS) films have a higher absorbance when compared to (PAH/PSS) and (QT/PSS) thin films, which may be directly related to the fact that, for the same pH, PEI has a lower degree of ionization than PAH and chitosan.

To better understand the growth of (PAH/PSS), (QT/PSS), and (PEI/PSS) thin films, the absorbance values at 195 nm were represented as a function of the number of bilayers (Figure 3d). The growth of all these films is linear, thus allowing for a critical control of their thickness, which is very important to avoid the sensor’s sensitivity loss due to the maximum range of the field lines created during the application of electrical potential. It should be noted that the absorbance at 195 nm, for both QT/PSS and PEI/PSS thin films, presents a similar behavior, indicating that the PSS adsorbed per area and bilayer is similar in the presence of chitosan or PEI molecules. This results in absorbance values higher than those of the PAH/PSS thin films, revealing that the PSS adsorbed per unit area in these films is about half when compared with those adsorbed on the QT or PEI layers. The linearity behavior can, however, only be verified after the formation of the first bilayers, as it initially depends on the roughness of the substrate, followed by electrostatic interactions and physical interactions associated with the pH of the solutions, the concentration, the adsorption time, the drying process, among other factors.

#### 3.1.2. Desorption Study on PBS Solution

**Figure 4:**
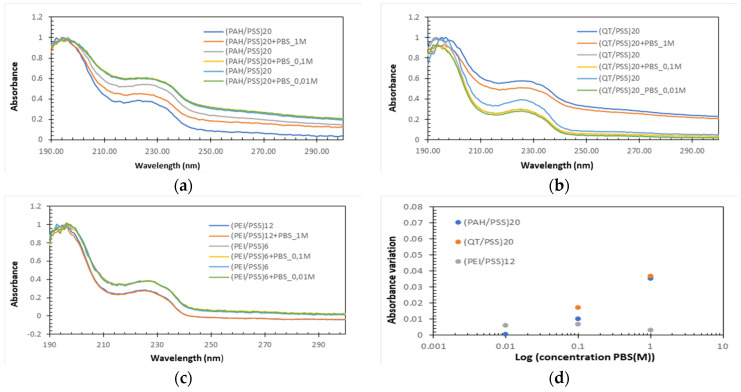
Normalized absorbance spectra of (**a**) (PAH/PSS), (**b**) (QT/PSS), and (**c**) (PEI/PSS) thin films when immersed for 1 h in PBS solution with 1 M, 0.1 M, and 0.01 M of concentrations. (**d**) Rate of change in absorbance (desorption rate) as a function of concentration.

Both the STC1A antibodies used to prepare the antibody layer and the antigen are dissolved in PBS; therefore, the stability of the prepared LbL films immersed in PBS must be evaluated. Moreover, Caroselli et al. [25] showed that the ideal time for protein A adsorption is about 1 h. As the protein A aqueous solutions were prepared in 0.1 M PBS, the behavior of the thin films in 1 M, 0.1 M, and 0.01 M PBS for 1 h was studied to verify whether there was desorption of the various bilayers formed. To analyze the desorption behavior, several (PAH/PSS)_20_, (QT/PSS)_20_, and (PEI/PSS)_20_ LbL films were prepared onto the quartz substrates and the spectra before and after the immersion during 1 h of the thin film in the PBS solution were measured. Several LbL films were used to analyze the desorption effect, the absorbance spectra of (PAH/PSS), (QT/PSS), and (PEI/PSS), and the normalization of thin films, as seen in Figure 4a–c, respectively. The normalization consisted in dividing the value obtained from the division of each of the spectra before and after desorption at different concentrations of PBS by the maximum absorbance of the spectrum at the same concentration before exposure to PBS. This normalization made it possible to relate the amount of desorption in each thin film with the concentration of PBS. To obtain the graph of Figure 3d, the difference in absorbance at 226 nm and 250 nm (∆ = abs_226nm_ − abs_250nm_) was calculated for each thin film before (∆_i_) and after exposure at 1 h PBS (∆f). Finally, the rate (∆_I_ − ∆_f_)/∆_i_ was calculated for each of the different PBS concentrations, obtaining the desorption behavior of the thin films as a function of the PBS concentration.

PAH/PSS thin films show a decrease in absorbance after exposure to PBS, whose difference increases as the concentration of PBS increases (Figure 4d). This could be directly related to the increased salt concentration in PBS. This increase allowed us to conclude that there was desorption and that the integrity of the thin films was affected. The behavior of (QT/PSS) was like that of (PAH/PSS) films. However, there was a greater desorption for a PBS concentration of 0.01 M. The values associated with 0.1 M and 1 M concentrations are slightly higher than those of (PAH/PSS) films. Finally, the PEI/PSS films show a small variance in the absorbance difference before and after exposure to different concentrations of PBS (Figure 4d) and the lowest difference in absorbance before and after exposure to PBS at concentrations of 0.1 M and 1 M than QT/PSS and PAH/PSS thin films. These results prove that, for the PEI/PSS thin films, the adsorption of protein A does not affect their integrity and that, for the QT/PSS and PAH/PSS films, there is a slight desorption that can affect the adsorption of protein A and, consequently, the final performance of the sensors.

### 3.2. Electrical Characterization of Cushions in PBS Aqueous Solutions

The solid supports with IDEs without deposited thin films and coated with (PAH/PSS)_5_/PAH, (QT/PSS)_5_/QT, and (PEI/PSS)_5_/PEI thin films were immersed in a volume of 3 mL of the different PBS solutions with concentrations 0.01 M, 0.1 M, and 1 M, at 23 °C, and the impedance spectra were measured. Electrical measurements were repeated twice for each concentration to verify the sensors’ reproducibility. As the salt concentration decreases, the more diluted the PBS, the higher the impedance value, which can be explained by the decrease in electrical conduction caused by the decrease in salt concentration. The desorption of the films was studied after 1 h in PBS to verify whether the integrity of the films was preserved in the interdigitated electrodes. From these results, it is noted that, when the (PAH/PSS)_5_/PAH and (QT/PSS)_5_/QT thin films are formed, regardless of the PBS concentration at which the measurement is being made, there is always an increase in impedance, since, by adding a new extra layer to the sensor, the effect of PAH as an insulating polyelectrolyte is more noticeable. In the case of chitosan, it is known that pure chitosan has an electrical conductivity of about 1.91 × 10^−4^ μS/cm, according to Marroquin et al. [38], and that the conductivity of the buffer solution, depending on the concentration, is on the order of mS/cm, so an increase in impedance was verified after the adsorption of the thin films. For the case of PEI, a decrease in impedance after the thin films’ adsorption is observed. It was also observed that, after 1 h in PBS (regardless of its concentration), the integrity of the films is completely preserved, making possible the adsorption of protein A without affecting them.

### 3.3. Adsorption Kinetics of Antigen on the Sensors’ Surfaces

To study the formation of the antibody layer with the adsorption of the protein A, BSA, and antibody onto IDEs uncovered and covered with thin films and consequent production of the sensors, we started by using solid supports with interdigitated electrodes without any film deposited to control the impedance of each of the solutions to be used in the production of the sensors and the comparison was made with the 0.1 M PBS solution to analyze whether each layer was adsorbed. With these results, one observed that, after the adsorption of protein A, there was an increase in impedance since the spectra presented higher impedance values for the same frequencies than the 0.1 M PBS solution. After the immersion of BSA, the spectrum gave higher impedance values than the protein A layer. Therefore, there is a further increase in impedance. However, after the antibody adsorption, the impedance spectrum had impedance values higher than those of PBS but lower than those of BSA and protein A layers, thus causing a decrease in impedance in relation to the two previously adsorbed layers. Finally, the immersion in the antigen solution leads to higher impedance values than those measured in PBS solution but lower than those of the antibody layer, BSA, and protein A, expecting a further impedance decrease after antigen adsorption.

The impedance of the various biomolecules composing the sensors was measured. When the thin films were used as cushions, there was an increase in impedance in sensor 1 and sensor 2 ((QT/PSS)_5_/QT) and a decrease in impedance in sensor 3 ((PEI/PSS)_5_/PEI). After protein A adsorption, there was an increase in impedance in all sensors, but to different degrees. With the adsorption of BSA and antibodies, the same trend in all sensors confirmed the above-mentioned results. These results show that it is possible to form the various desired layers and that all sensors can detect an antigen concentration of 5 × 10^−4^ M since there is always an impedance variation in relation to the previously adsorbed layer. 

**Figure 5:**
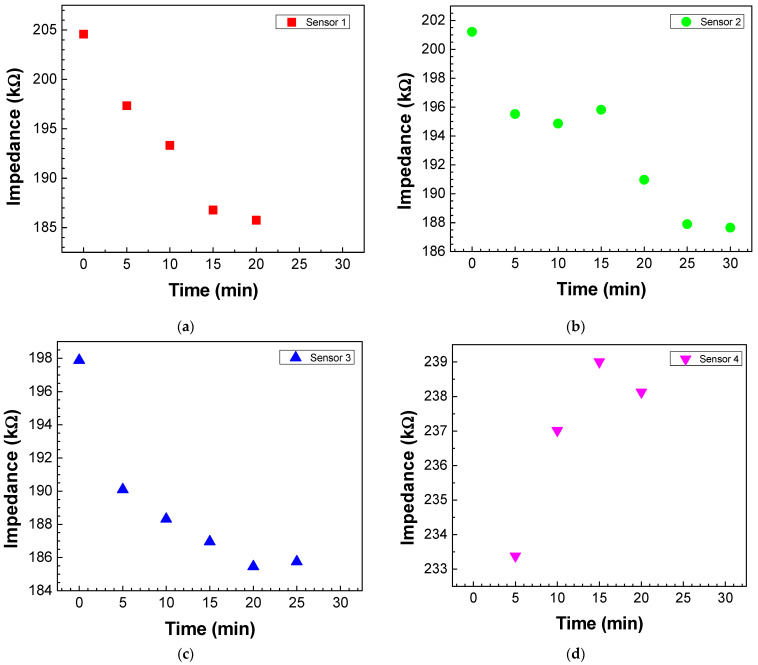
Adsorption kinetics curves of antigen molecules on the (**a**) sensor 1, (**b**) sensor 2, (**c**) sensor 3, and (**d**) sensor 4. The kinetics curves were achieved by plotting the impedance values measured at 1 Hz as a function of the immersion time in the antigen solution with a concentration of 5 × 10^−4^ M.

To understand when saturation occurs, adsorption kinetics curves were obtained by plotting the impedance measured at 1 Hz (the frequency whose impedance variation is greater) for the different sensors as a function of the immersion time in the antigen solution with a concentration of 5 × 10^−4^ M. The exposure time of sensors 1, 2, and 3 (Figure 5a–c, respectively) in the antigen solution leads to an impedance decrease in these sensors. However, sensor 1 saturates after 20 min, and sensors 2 and 3 after 25 min. Sensor 4 (Figure 5d) also saturated after 20 min, but there was an impedance increase instead of an impedance decrease until saturation.

### 3.4. Testing of Sensors at Different Antigen Concentrations

**Figure 6:**
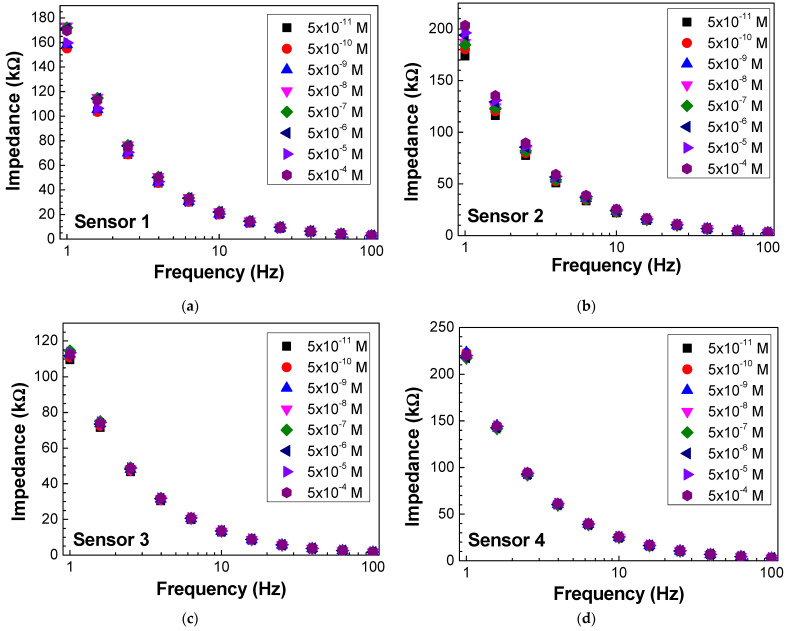
Impedance spectra of (**a**) sensor 1, (**b**) sensor 2, (**c**) sensor 3, and (**d**) sensor 4 when immersed in different concentrations of antigen.

**Figure 7:**
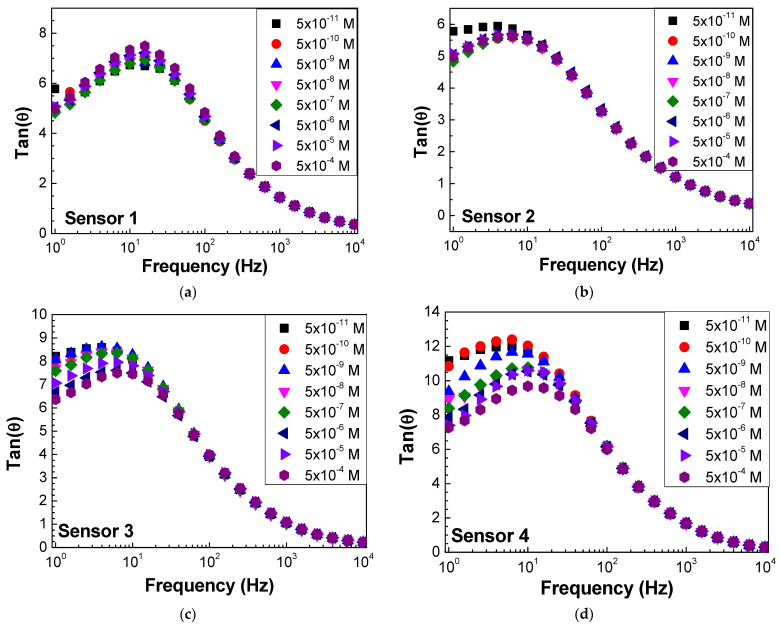
Loss tangent spectra of (**a**) sensor 1, (**b**) sensor 2, (**c**) sensor 3, and (**d**) sensor 4 when immersed in different concentrations of antigen.

Impedance and loss tangent spectra were measured when the sensor was immersed in the antigen solutions at different concentrations, as seen in Figure 6 and Figure 7, respectively. Both impedance and loss tangent spectra change with the antigen concentration. The major changes are observed for low frequencies for all the sensors.

**Figure 8:**
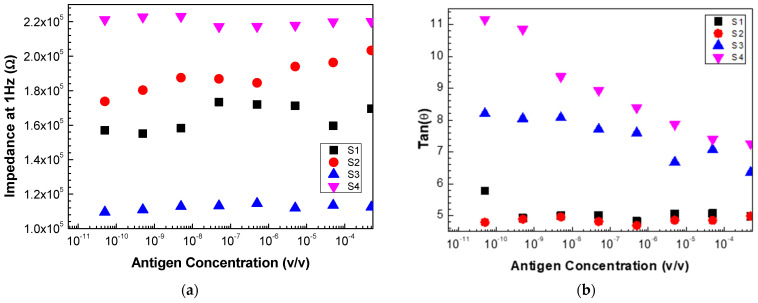
Evolution of (**a**) impedance and (**b**) loss tangent at 1 Hz measured with sensor 1 (S1), sensor 2 (S2), sensor 3 (S3), and sensor 4 (s4) as a function of the antigen concentration.

To verify the response of each of these sensors to different antigen concentrations, the impedance and the loss tangent values measured at 1 Hz were plotted for the various sensors (Figure 8a,b). Sensors 2 and 3 presented a more linear response, while the other sensors did not have a clear evolution with the concentration. However, analyzing the response of the sensors when the loss tangent values are considered for the frequency values indicated above, as seen in Figure 8b, one can conclude that sensors 3, 4, and 2 exhibit a linear response with the increase in concentration.

These results allow us to conclude that, for the PCA, for each of the individual sensors and the electronic tongue (the set of all sensors), it is essential to consider a specific range of frequencies and not just a fixed frequency and, instead of the impedance values, the loss tangent should be considered.

### 3.5. Electronic Tongue Performance

**Figure 9:**
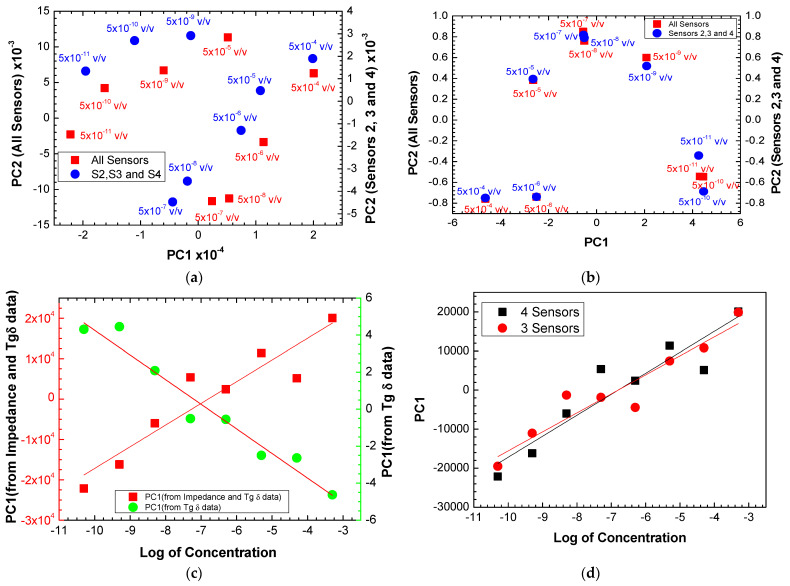
PCA plot using (**a**) impedance and tangent loss spectra and (**b**) only tangent loss spectra for all the sensors and for sensors 2, 3, and 4, measured when the sensors were immersed in different antigen concentrations. (**c**) Evolution of the PC1 values calculated with the impedance + loss tangent data and only loss tangent data measured with all the sensors as a function of the logarithm of the antigen concentration. (**d**) Evolution of the PC1 values calculated with the impedance + loss tangent data measured with all the sensors and with sensors 2, 3, and 4 as a function of the logarithm of the antigen concentration.

From the PCA of all the sensors using variables’ impedance and tangent loss spectra as well as sensors 2, 3, and 4 using only variable tangent loss spectra (Figure 9a,b), the PC1 values show growth and decay trends with the logarithm of antigen concentration. The PC2 values have no relationship with the antigen concentration in all the cases. In both cases, the electronic tongue presents a linear trend, with the various sensors capable of distinguishing different concentrations. 

To compare the behavior of the electronic tongues using all the sensors and only sensors 2, 3, and 3, the PC1 values achieved from the PCA plots in Figure 9a were plotted as a function of the logarithm of the antigen concentration, as seen in Figure 9d. Both plots present similar trends with a straight line fitting the data. The slopes obtained from $\frac{\Delta \mathrm{PC}1}{\Delta \log\left(C\right)},\;$were 5371 ± 820 and 4863 ± 634 per decade of antigen concentration (*C*), meaning the sensitivity of the electronic tongue with the four sensors is higher than the best sensors (sensors 2, 3, and 4). However, the uncertainty is also higher in the case of the electronic tongue with all sensors (15%) than the electronic tongue with the best sensors (13%). Table 2 summarizes the sensitivity and resolution parameters of electronic tongues formed by all sensors, by the three best and individual sensors. The resolution of the sensors is the smallest amount of concentration that they can discriminate and was obtained through the following expression: $\Delta \log\left(C\right)=\frac{Sensitivity\;uncertainty}{Sensitivity}=\log(C)-\log\left(C_{i}\right)$, where $C_{i}\;$corresponds to the value of the lowest concentration used and the resolution is given by the calculated *C*. By analyzing the data in Table 2, one can confirm that the use of the array of sensors (electronic tongue) makes the results more robust than using just the best sensor (sensor 2), as the uncertainty associated with sensitivity is smaller and the resolution values are practically the same. It is also concluded that sensors that use layers of polyelectrolyte thin films (sensors 1, 2, and 3) have greater sensitivity compared to sensor 4 without polyelectrolyte thin films. Table 3 summarizes the literature results of sensors for detecting prostate and ovarian cancer using antibodies and immobilizing antibodies on the sensors’ surfaces for comparison with the results achieved in this work.

## 4. Conclusions

In this work, we developed a prototype of an electronic tongue based on thin films of Stanniocalcin-1 protein antibodies to diagnose prostate cancer. To this end, a sensor array was developed to detect STC1 protein antigen concentrations between 5 × 10^−11^ and 5 × 10^−4^ M.

The electronic tongue sensors were based on thin films of different materials deposited on sensor devices with interdigitated electrodes using layer-on-layer deposition and self-assembly techniques. These techniques guarantee that the deposited layers have thicknesses in the order of nanometers. Four types of sensors were produced in which solid supports with interdigitated electrodes were coated with thin films of PAH/PSS, QT/PSS, and PEI/PSS, and on each of these sensors, protein A was deposited on the film’s surfaces, followed by the blocker BSA and finally the STC1 antibodies were deposited. Finally, the fourth sensor did not have thin films of polyelectrolytes but had the thiolate protein A, the surface blocker, and the antibodies.

The growth of PAH/PSS, QT/PSS, and PEI/PSS films was analyzed by UV–vis spectroscopy, and the spectrum demonstrated the formation of the various bilayers of thin films of polyelectrolytes and that the growth of these films is proportional to the number of deposited bilayers. This guarantees control of the thickness of the layers. The desorption of thin films was also studied at different concentrations of PBS (1 M, 0.1 M, and 0.01 M) since, to produce solutions of other biomolecules, it was necessary to use PBS to guarantee a stable pH, thus increasing their efficiency. It was concluded that, for PAH/PSS thin films, there was a desorption and that it was greater the greater the concentration of PBS when exposed for 1 h to PBS solution. There was a lower desorption for the PEI/PSS films than the other thin films, which remained constant regardless of the PBS concentration.

Impedance spectroscopy was also used to study the formation of thin films of polyelectrolytes on sensor devices with interdigitated electrodes. It was concluded that, for the PAH/PSS and QT/PSS films after the adsorption of the thin films, there was an increase in impedance, proving that these compounds have low conductivity and that, for thin films of PEI/PSS, there was a decrease in impedance.

All sensors detected an antigen concentration of 5 × 10^−4^ M and the mean saturation time was about 20 min. All sensors could distinguish different antigen concentrations, although this distinction was more noticeable for certain (low) frequencies.

The data obtained by the different sensors were statistically analyzed using the principal component analysis (PCA) method. It proved capable of separating the sensors and their respective concentrations with variability in the data of 99%. The sensor array (electronic tongue) provided exciting results, showing a positive evolution as the antigen concentration increased. The electronic tongue formed by the four sensors had a sensitivity of 5371 ± 820 and the one formed by the three best sensors a sensitivity of 4863 ± 634. This shows that, for laboratory or medical purposes, with a lower error rate, it would be more advisable to use the electronic tongue formed by the three best sensors. This result was also compared with the best of the sensors, and it was concluded that the use of the electronic tongue in fact produces more robust and credible results. We conclude that this prototype can advance to the calibration phase with actual samples. Including more sensors to develop the prostate cancer prototype may be, however, necessary.

## Figures and Tables

**Table 1:** Values of the degree of ionization of PAH, PEI, and chitosan as a function of pH.

Polyelectrolyte	pH Range	Degree of Ionization
PAH	5–6	0.8 [33]
PSS	5–6	0.65 [36]
Chitosan	4–6	1 [37]

**Table 2:** Sensitivity and resolution for each sensor, the electronic tongue formed by the 4 sensors and the electronic tongue formed by the 3 best sensors calculated from the PCA.

Sensor	Sensitivity (per Concentration Decade)	Resolution (M)
S1	2400 ±1422	${1.96\times 10}^{-10}$
S2	4790 ±644	${6.81\times 10}^{-11}$
S3	626 ± 254	${9.89\times 10}^{-10}$
S4	−258 ± 422	${1.16\times 10}^{-12}$
ET with 4 Sensors	5372 ±820	${7.11\times 10}^{-11}$
ET with 3 Sensors *	4863 ± 634	${6.75\times 10}^{-11}$

* Sensors 2, 3, and 4.

**Table 3:** Comparison of sensors for detecting prostate and ovarian cancer using antibodies and immobilizing antibodies on the sensor’s surface.

Study	Immobilization Technique	Transduction Method	Advantages	Disadvantages	Sensitivity/Resolution
Lectin microarray [39].	Lectin and antibodies immobilized using biotin affinity	Fluorescent labels	Fast, minimal reagent consumption, good stability	Low sensitivity and specificity, randomly oriented antibodies	Linear range of 0.5–10μg/mL
Rapid test for lectin–glycan detection [40].	Lectin covalently immobilized on copper/nickel/gold electrodes on a PCB board	Impedance spectroscopy (EIS)	Fast test (80 s) and high sensitivity	Use of nanoparticles to amplify the signal due to the presence of noise	Linear range of 0.5 ng/L–50 µg/L with a detection limit of 0.5 ng/L
Glycosylated biomarker sensors [41].	Use of gold nanoparticles on electrodes	Amperometry	Increased electron transfer, biocompatibility, ease of lectin immobilization	Use of gold nanoparticles, lacks validation in a clinical context	Detection limit of 0.1 μM
Quartz microbalance sensor [42].	Immobilization by physical adsorption	Change in resonance frequency	Low cost, high speed, simple instrumentation, use of polyclonal antibodies	Random orientation of antibodies, which can decrease sensitivity, dependence on the dipole moment of the antibody and the charge on the support surface, and time-long answer	Linear range of 0.5–10μg/mL
Photonic sensor [25]	Use of protein A	Wavelength changes	Orientation of antibodies ensuring good sensitivity, protein A surface binding by physical adsorption, protein A—reversible antibody binding. Quick, cheap, and easy immobilization.	Extensive adsorption of protein A can lead to denaturation and loss of antibody functionality	Detection of 1 ng/mL
This work	Use of Protein A and Stanniocalcin-1 protein antibodies	Impedance spectroscopy	The developed sensors can be used with other sensors that allow for matrix detection.	Other several sensors will be necessary for prostate cancer detection	${4863\pm 634\;*/6.75\times 10}^{-11}$ M

* Sensors 2, 3, and 4.

## Data Availability

Data are contained within the article.
